# The simply modified intrascleral fixation using round flange (SMURF) technique for intrascleral intraocular lens fixation

**DOI:** 10.1038/s41598-021-81924-y

**Published:** 2021-02-16

**Authors:** Soa Kim, Jee Taek Kim

**Affiliations:** 1 Department of Ophthalmology, Suwon Hospital, Gyeonggi Provincial Medical Center, Suwon, South Korea; 2 Department of Ophthalmology, College of Medicine, Chung-Ang University Hospital, 102 Heukseok-ro, Dongjak-gu, Seoul, 06974 South Korea

**Keywords:** Eye diseases, Lens diseases, Vision disorders

## Abstract

We describe a simply modified intrascleral fixation using round flange (SMURF) technique and report the clinical outcomes of the surgery. Forty-one eyes of 41 consecutive patients, with intraocular lens (IOL) dislocation, crystalline lens subluxation, and zonular weakness, who underwent surgery using the SMURF technique were included. The modified technique included the use of a conventional 27-gauge needle, a non-bent needle, oblique sclerotomy, direct threading of the leading haptic, and simple placement of the following haptic. IOLs were successfully placed and showed good centring. There were no cases of wound leakage or hypotony during the early postoperative period. Postoperative complications included vitreous haemorrhage in one eye (2.4%), intraocular pressure elevation in one eye (2.4%), and iris capture in six eyes (14.6%). There were no cases of postoperative retinal detachment, cystoid macular oedema, endophthalmitis, or IOL dislocation during the follow-up period. We proposed a few modifications in the intrascleral flanged technique for IOL fixation. The modified technique is a simple, easy, and minimally invasive procedure for successful IOL intrascleral fixation.

## Introduction

Implantation of intraocular lenses (IOLs) is important in eyes with insufficient zonular or capsular support. Open-loop anterior chamber (AC), iris-claw, and scleral-sutured posterior chamber (PC) IOLs are used to correct aphakia in such eyes^1^. The Ophthalmic Technology Assessment Committee of the American Academy of Ophthalmology performed a literature review of the surgical procedures, and concluded that all the procedures are comparably safe and effective for correcting aphakic states^1^. However, all of these procedures are associated with complications. Specifically, AC IOLs may cause endothelial cell loss, corneal decompensation, and angle compromise^2,3^, whereas iris-fixated IOLs may cause intraocular inflammation, cystoid macular oedema, and endothelial cell loss^4–6^. The scleral-sutured PC IOL is a well-established, reliable method for IOL implantation^1,7–9^; however, some complications may occur, including suture erosion and degradation, which can lead to IOL dislocation^10^. Another major drawback of this method is the prolonged surgical time.

Recently, the sutureless intrascleral fixation technique for IOL implantation has become popular^11–16^. Akimoto et al. reported an intrascleral fixation technique using a modified catheter needle to externalise IOL haptic and a 30-gauge thin-wall needle to create scleral tunnels^17^. Rensburg et al. reported a reinsertion technique of the haptic tip into the vitreous cavity after externalisation to reduce haptic exposure^18^. Yamane et al. described a trans-conjunctival sutureless intrascleral fixation technique with a flanged haptic ending^19^. An advantage of the intrascleral fixation technique is its simplicity, as compared to the transscleral fixation technique^19^. However, it requires additional corneal incisions, a specially designed 30-gauge thin-wall needle (TSK ultra-thin wall needle; Tochigi Seiko, Tochigi, Japan), and retinal forceps, which can be considered as a drawback. There is also the risk of retinal injury, as well as the risk of dropping the IOL into the vitreous cavity during the procedure^14,15^. Furthermore, Yamane’s intrascleral fixation technique, which requires insertion of the IOL haptics into the needle, is technically difficult to perform^19^. Thus, some modified techniques have been described^14–16,18^.

In this report, we propose some modifications to the procedure for simplicity and improved safety. These modifications provide several advantages in terms of risk of retinal injuries and reduced number of procedural steps, corneal wounds, and use of intraocular forceps. We termed this as the ‘simply modified intrascleral fixation using round flange (SMURF)’ technique, given that these modifications were designed to eliminate technical difficulties. Thus, this study aimed to describe this surgical technique and report the surgical outcomes.

## Methods

### Subjects

This retrospective interventional, longitudinal study was approved by the Institutional Review Board Committee of Chung-Ang University Hospital, Seoul, South Korea, and adhered to the tenets of the Declaration of Helsinki. Medical records of consecutive patients who underwent the SMURF technique for IOL fixation at the retina clinic of Chung-Ang University Hospital between 1 October, 2016, and 30 December, 2018, were analysed. Informed consent was obtained from all patients for the surgery and use of ocular images for publication.

The inclusion criteria were eyes with inadequate capsular support as follows: eyes with (1) an aphakia state, (2) dislocation of the one-piece IOL, (3) severe subluxation or dislocation of the crystalline lens, (4) intraoperative rupture of the posterior capsule due to extension of a radial tear of the anterior capsule, and (5) zonular weakness. All of the patients who underwent surgery using the SMURF technique were included in the study. Postoperative complications were evaluated during the follow-up periods. Surgical outcomes were analysed at 12 months, postoperatively.

Comprehensive ophthalmologic examinations were performed before and after surgery, and included measurement of uncorrected visual acuity (UCVA), best-corrected visual acuity (BCVA), intraocular pressure, and refractive error. Slit-lamp examination, fundus examination and photography, and specular microscope examination were performed. The angle of the IOL tilt was measured using swept-source optical coherence tomography (SS-OCT; DRI Triton OCT, Topcon, Tokyo, Japan) 1 month after the surgery. Horizontal and vertical B-scan images with a length of 16 mm were captured. The B-scan image was exported in a tagged image file format, and the angle between the iris plane and horizontal axis of the IOL was analysed using ImageJ software (National Institutes of Health, Bethesda, MD, USA). The average IOL tilt in the vertical and horizontal planes was defined as the mean IOL tilt.

### Surgical technique and surgical points

A complete vitrectomy using a standard 3-port system was performed under retrobulbar or general anaesthesia. The flanged intrascleral fixation surgery was performed with the following modifications without any assistance from a surgical assistant.

#### Meridian or left-sided corneal incision

**Figure 1:**
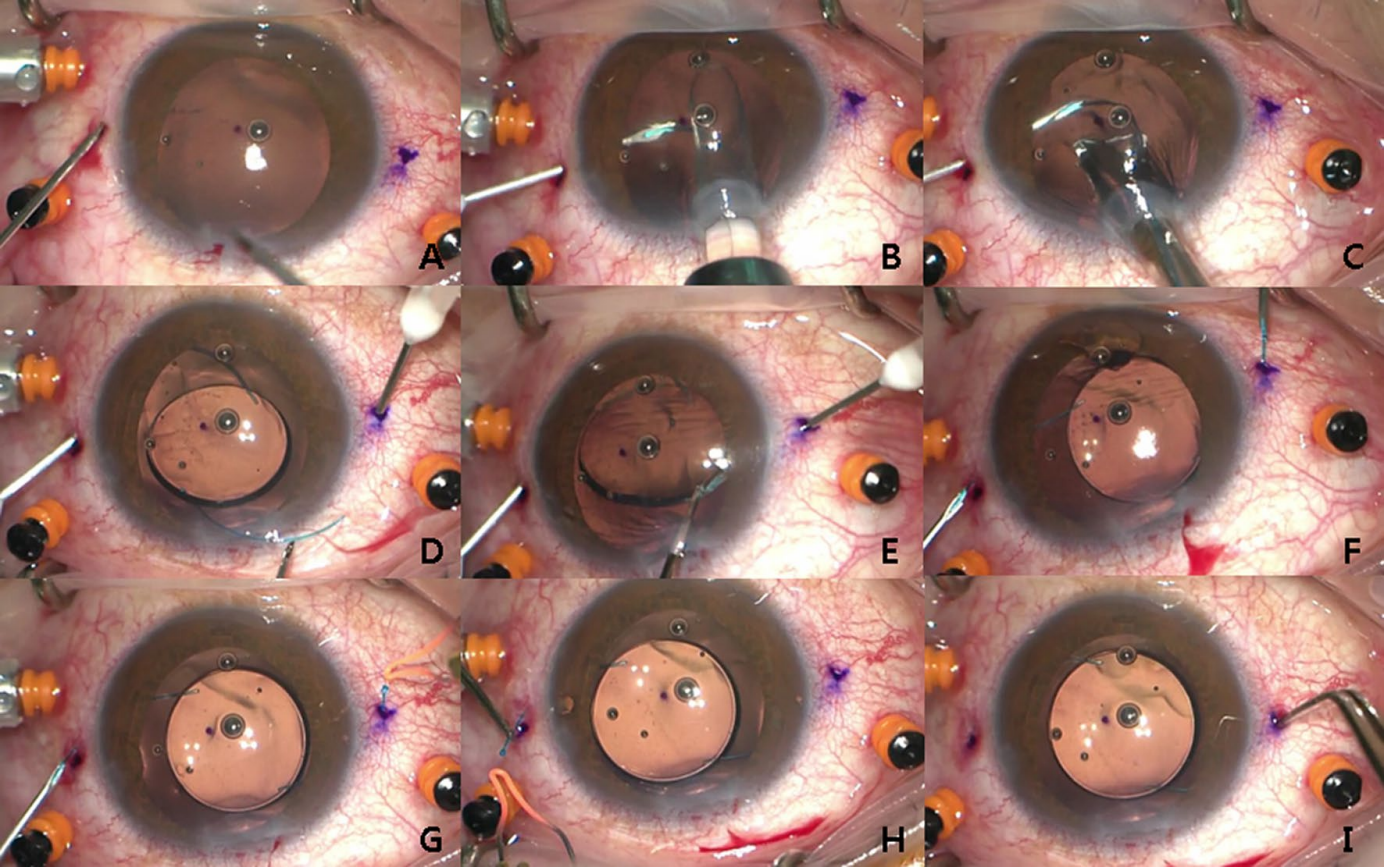
Intraoperative photographs showing the Simply Modified Intrascleral Fixation Using Round Flange (SMURF) technique. (**A**) After a pars planar vitrectomy, a 2.75 mm clear corneal incision was made in the 12 o’clock to 1 o’clock direction for intraocular lens (IOL) insertion. An oblique sclerotomy was performed 2.0 mm from the limbus using a conventional 27-gauge needle. (**B**) A leading haptic was directly placed into the lumen of the first needle without the use of forceps after the leading haptic was injected into the anterior chamber. (**C**, **D**) The entire optic was injected into the anterior chamber, leaving the trailing haptic at the corneal wound, after placement of the leading haptic into the first needle. (**D**) A second oblique sclerotomy was performed with the same technique and the same conventional 27-gauge needle. (**D**, **E**) The trailing haptic was grasped 3–4 mm from the end of the haptic with intraocular forceps and was simultaneously inserted into the anterior chamber and the lumen of the second needle. (**F**) The haptics were externalised with the two 27-gauge needles. (**G**, **H**) The ends of the haptics were cauterised to create flanges. I. Both flanged ends of the haptics were buried intrasclerally. The images showed good centring of the IOLs after the procedures.

**Figure 2:**
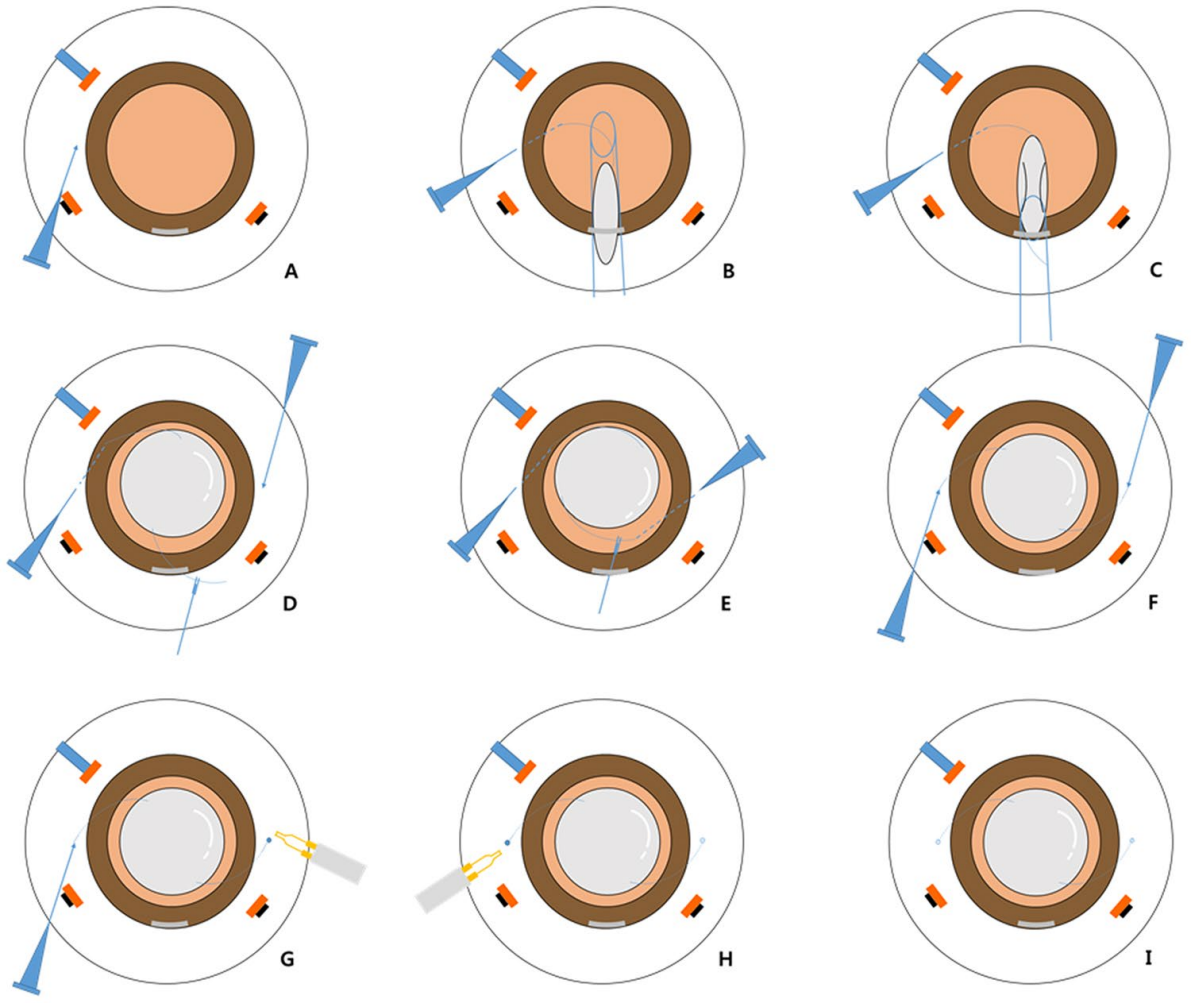
Step-by-step schematic illustration of the Simply Modified Intrascleral Fixation Using Round Flange (SMURF) technique. (**A**) An oblique (with an angle of 30° with respect to the limbus) angled (less than 5°–10° with respect to the surface of the iris) sclerotomy was performed 2.0 mm from the limbus using a conventional 27-gauge non-bent needle. A clear corneal incision should be made in the 12 o’clock to 1 o’clock direction before sclerotomy. (**B**) An intraocular lens (IOL) injector was introduced into the anterior chamber (AC), and IOL was injected slowly. When the tip of the leading haptic was initially shown in the AC, the left hand was moved from the screw mechanism plunger to the 27-gauge needle. The tip of the leading haptic was directly threaded into the 27-gauge needle and the angle of the bevel of the 27-gauge needle and tip of the haptic were adjusted. (**C**) The entire optic was injected into the AC after the anchoring of the leading haptic. At this point, the trailing haptic should be left at the corneal incision site to prevent drop of the IOL. (**D**) A second oblique sclerotomy was performed. At this point, the surgeon should confirm that the direction of both the 27-gauge needles is parallel and symmetrical. If not, the angle of the second sclerotomy should be adjusted. Then, the trailing haptic should be grasped with retinal forceps at the distal one-third point of the haptic. (**D**, **E**) The trailing haptic should be pushed into the AC using retinal forceps with the left hand and threading simultaneously with the second needle. If the trailing haptic could not be well-pushed into the AC, the grasping point should be adjusted. If the grasping is distal, the haptic does not easily insert into the AC. Grasping the proximal part of the haptic leads to difficulties in threading the haptic into the needle, although the haptic could be easily inserted into the AC. When using intraocular forceps on the left hand, the right hand should grasp the second needle to be threaded simultaneously. (**F**) Both haptics were externalised. (**G**) The tip of the trailing haptic was cauterised to create flanges. (**H**) The ends of the leading haptics were cauterised. I. Both flanged ends of the haptics were buried intrasclerally.

A 2.75-mm clear corneal incision for IOL insertion or removal was made in the 12 o’clock –1 o’clock direction (left side of the median line) to make it easier to use the left hand (Figs. 1A, 2A). In cases of IOL subluxation, all IOLs were cut and removed through a 2.75-mm corneal incision because the manipulation of the haptic of the dislocated IOL within the AC was difficult to thread into the needle as in the original Yamane’s technique.

#### Use of non-bent conventional 27-gauge needles

Non-bent conventional 27-gauge needles were used because an asymmetrical bend of the needle could affect the asymmetrical angle of the sclerotomy and consequently lead to IOL tilting. It is possible, but difficult, to insert IOL haptics into 30-gauge thin-wall needles due to the small lumen size. Thus, the threading of haptics into the 30-gauge needle may occasionally cause haptic deformation. Therefore, we used a 27-gauge needle because it has a larger lumen through which IOL haptics can be inserted easily. Additionally, non-bent needles were used in this study. The asymmetrical bend of the needle may affect the angle of the bevelled sclerotomy, and thus the asymmetrical angle of the sclerotomy might affect the position and tilting of the intrasclerally fixed IOL.

#### Oblique sclerotomy

An oblique (with an angle of 30º with respect to the limbus) angled (less than 5–10º with respect to the surface of the iris) sclerotomy was performed to reduce retinal damage (2.0 mm behind the limbus) (Figs. 1A; 2A; see Supplementary video 1).

#### Direct threading of the leading haptic

The tip of the leading haptic was directly inserted from the IOL injector into the lumen of the needle without additional paracentesis, use of intraocular forceps, or help from a surgical assistant (Figs. 1B, 2B; see Supplementary video 2). When the leading haptic emerges out of the injector into the AC, the tip of the haptic can be directly inserted into the lumen of the needle (with the bevel facing the haptic). After placement of the haptic tip into the lumen, the leading haptic can be injected into the AC slowly with a twist-type plunger, using the left hand, while the right hand holds the injector shaft. Then, the haptic can be threaded deeply (in a slow manner) into the needle using both hands and a step-by-step repeated procedure.

#### Threading of the trailing haptic

The IOL was injected after placing the leading haptic into the needle (Figs. 1C, 2C). A second oblique sclerotomy was created (Fig. 1D; Fig. 2D; see Supplementary video 3). The trailing haptic could be relatively easily and gently threaded with a one-time use of intraocular forceps (Alcon 23-gauge Advanced DSP tip #723.45, GRIESHABER asymmetrical disposable forceps with the GRIESHABER REVOLUTION handle) using the left hand without additional paracentesis or the bi-manual handshake technique (Figs. 1D, E, 2D, E; see Supplementary video 4).

#### Externalisation and cauterisation

The placed haptics within the 27-gauge needles were externalised onto the conjunctiva (Figs. 1F, 2F; see Supplementary video 5). The haptic ends were cauterised using a hand cautery device (Accu-Temp Cautery; Beaver Visitec, Waltham, MA) to make a flange with a diameter of 300 µm (Figs. 1G, H, 2G, H; see Supplementary video 6). Considering the outer diameter of the 27-gauge needle (0.4 mm), 1.5–2.0 mm of the IOL haptic was flanged to secure the IOL. The flanges were inserted and fixed into the sclera without exposure (Figs. 1I, 2I; see Supplementary video 6).

#### Iridotomy and wound suture

A peripheral iridotomy was performed in the superior nasal quadrant using a vitrectomy cutter after miosis to prevent IOL capture by the iris. Corneal incision was sutured with #10-0 Nylon in all cases. All sclerotomies which tended to leak were also sutured with #8-0 Vicryl.

## Results

### Baseline characteristics

In total, 41 patients (41 eyes) underwent the surgery and were included in this study. The mean age of the patients was 72.3 ± 11.26 years (range = 27–86), and the mean axial length was 24.49 ± 1.52 mm (range = 22.06–26.44) at the time of surgery. The mean duration of follow-up was 13.61 ± 7.4 months (range = 1–24). The numbers of patients followed-up for 1, 3, 6, and 12 months were 41, 34, 33, and 32, respectively.

The most common cause for surgery was idiopathic IOL subluxation (16 eyes); the second most common cause was idiopathic crystalline lens subluxation (10 eyes). Other causes included crystalline lens subluxation due to intraoperative posterior capsule tear (8 eyes), and traumatic (4 eyes) or idiopathic (3 eyes) zonular weakness. The patients’ characteristics are shown in Table 1.

**Table 1:** Baseline characteristics and preoperative and postoperative clinical profiles of the patients included in this study.

Characteristics	Baseline	12 months	P*
Total number of eyes/ patients	41/41	32/32	
Age, years (range)	72.3 ± 11.26 (27–86)	71.5 ± 13.27 (27–86)	0.16
Sex (male/female), n	33/8	26/6	
**Lens status and cause, n (%)**			
IOL dislocation	16 (39.0%)	14(43.8%)	
Idiopathic	16 (39.0%)	14(43.8%)	
Traumatic	0 (0%)	0 (0%)	
Crystalline lens subluxation	18 (43.9%)	11 (34.3%)	
Idiopathic	10 (24.4%)	8 (25%)	
Traumatic	0 (0%)	0 (0%)	
Intraoperative PC tear	8 (19.5%)	3 (9.4%)	
**Aphakia**	0 (0%)	0 (0%)	
Zonular weakness	7 (17.1%)	7 (21.9%)	
Idiopathic	3 (7.3%)	3 (9.4%)	
Traumatic	4 (9.8%)	4 (12.5%)	
UCVA, logMAR UCVA, Snellen VA	0.96 ± 0.81 20/200	0.3 ± 0.14 20/40	0.002
BCVA (logMAR) BCVA Snellen VA	0.40 ± 0.65 20/50	0.06 ± 0.06 20/25	0.006
Refractive error	5.17 ± 7.37	− 1.27 ± 1.25	< 0.001
Endothelial cell count per mm^2^	2628.4 ± 783.7	2312 ± 612.6	0.02

Data are presented as the mean ± standard deviation, n (%), or the range. *Independent t-test.

BCVA, best-corrected visual acuity; IOL, intraocular lens; logMAR, logarithm of the minimum angle of resolution; PC, posterior capsule; UCVA, uncorrected visual acuity.

### Surgical outcomes

**Figure 3:**
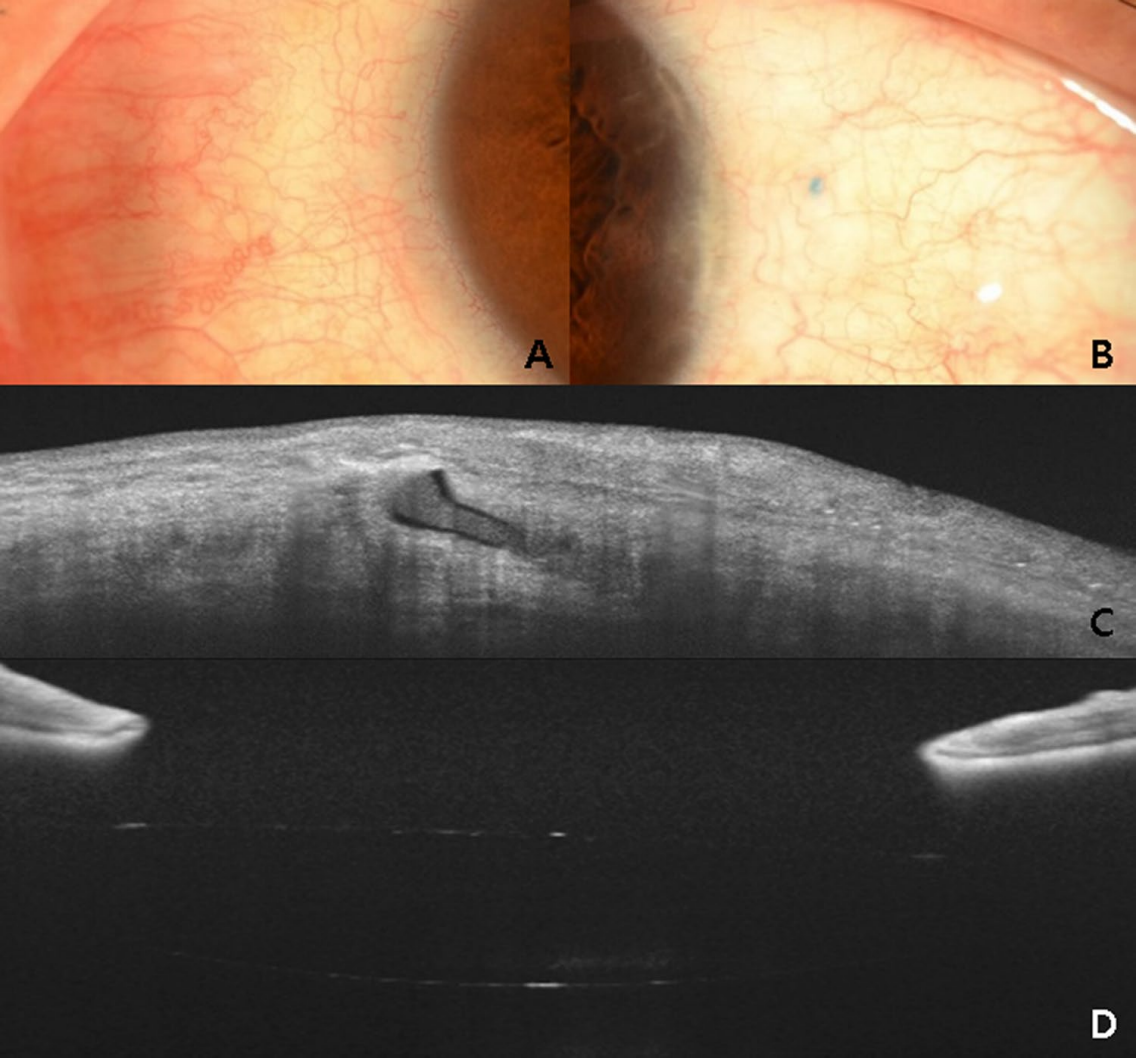
Postoperative findings of the flanged endings and intraocular lens six months after surgery. The uncorrected and best-corrected visual acuities were 20/33.3 (Snellen visual acuity) and 20/20 (Snellen visual acuity), respectively. (**A**, **B**) An image of the anterior segment showing intrascleral flanged endings without conjunctival erosion or flange exposure. © Optical coherence tomography (OCT) image of the anterior segment showing intrascleral flanged endings of the haptic shown in panel (**B**). The largest diameter of the flange was 300 µm. (**D**) OCT images showing no tilting of the intraocular lens.

The IOL was well centred and fixated without conjunctival erosion or haptic exposure (Fig. 3). Table 1 shows the refractive outcomes of the included patients. The mean refractive difference between the preoperative predictive value and postoperative value was + 0.09 ± 0.9 dioptres (range = − 0.96 ± 1.05), and the IOL-induced astigmatism was -0.20 ± 0.41 dioptres (range = − 1.25 – 0) at 12 months postoperatively. The mean endothelial cell count per mm^2^ was 2628.4 ± 783.7 (range = 1272–3610) before surgery and decreased to 2312 ± 612.6 (range = 1120–2532) at 12 months after surgery (*P* = 0.02). The mean IOL tilt was 3.5° ± 3.2°. Two models of three-piece IOLs were used in this study; the Tecnis ZA 9003 Aspherical IOL (AMO Johnson & Johnson Vision, Inc, Abbott Park, Illinois, USA) and Sensar AR 40e spherical IOL (AMO Johnson & Johnson Vision) were used in 12 and 20 eyes, respectively. However, visual outcomes were not significantly different.

### Surgical complications

Postoperative complications are shown in Table 2. The most common complication was intermittent partial iris capture, observed in six eyes (14.6%). The iris capture was released using mydriatic eye drops and enlargement of the iridotomy site. Another complication was vitreous haemorrhage (one eye; 2.4%) caused by intraoperative bleeding from the ciliary body or the iris root. This complication resolved spontaneously 1 week after surgery. Transiently increased intraocular pressure was observed in one eye (2.4%) on postoperative day 1, and this normalised after the use of eyedrops. Residual viscoelastic or dispersed iris pigments may have caused intraocular pressure elevation. There were no other significant complications (i.e., postoperative hypotony, cystoid macular oedema, IOL dislocation, haptic exposure, retinal detachment, or endophthalmitis) during the 12-month follow-up period. No patients required a second operation or repositioning of the flanged endings.

**Table 2:** Postoperative complications.

Complications	No. of eyes (%)
**Early complications within 1 week after surgery**	
Vitreous haemorrhage	1 (2.4%)
Hypotony	0 (0%)
IOP elevation	1 (2.4%)
**Late complications between 1 week and 12 months after surgery**	
Iris capture of IOL	6 (14.6%)
Retinal detachment	0 (0%)
Cystoid macular oedema	0 (0%)
Endophthalmitis	0 (0%)
IOL dislocation	0 (0%)

IOL, intraocular lens; IOP, intraocular pressure.

## Discussion

For decades, many surgical techniques have been proposed for IOL implantation in eyes with inadequate capsular support. The transscleral suture technique using 10-0 prolene is a conventional, reliable method; however, the time-consuming nature of the technique and the long learning curve have prompted several technical advancements. In 2007, Gabor et al. first adapted the technique for sutureless intrascleral fixation with haptic externalisation using 25-gauge end-gripping forceps^11^. The technique was less time-consuming and comparatively easier than the conventional technique. Thereafter, several modified techniques have been proposed. In 2008, Agarwal et al. proposed the glued-IOL technique to stabilise IOL haptics beneath the scleral flap^12^. In 2009, Rodriguez-Agirretxe et al. described a 25-gauge needle-guided haptic externalisation technique^13^. Further, in 2017, Yamane et al. proposed a flanged intrascleral IOL fixation technique that utilised cauterisation to create flanged ends of the haptics^19^.

Yamane’s technique was superior to previous methods as it required less surgical time and produced less haptic damage^20^. However, the double needle technique has a few drawbacks. First, it is difficult to manipulate the intraocular forceps, especially when the leading or following haptics are inserted into the lumen of the needles using forceps, because the retinal forceps that were designed for the vitreo-retinal procedure have to be manipulated in the plane horizontal to the iris. Second, haptic threading without haptic deformation is difficult when the haptics are grasped with the intraocular forceps and inserted into the lumen of the ultra-thin wall 30-gauge needle. Third, there is a risk of retinal injury or surrounding tissue damage. Placement of the needle tip within the vitreous cavity may result in a higher risk of retinal injury.

Therefore, we made a few modifications to reduce technical difficulties and potential tissue injury. First, a conventional 27-gauge needle was used. The TSK 30-gauge ultra-thin wall needle was originally developed to reduce injection resistance for cosmetic filler injections. However, occasionally, haptic deformity may result from threading the IOL haptic into a 30-gauge needle. Moreover, the 30-gauge needle is not commercially available in some countries. A 27-gauge needle has a larger lumen. Thus, IOL haptic can be easily inserted without haptic damage.

Second, we believe that the non-bent needle is favourable to prevent IOL tilting. The asymmetrical bend of the needle may result in the asymmetrical angle of the sclerotomy and tilting of the intrascleral-fixated IOL. Further, the bent needle and the bevel of the needle tended to rotate when the needle is not grasped. Therefore, we do not prefer the bent needle; however, sunken eyes or eyes with a small lid fissure sometimes require a bent needle. Moreover, the left eye more commonly requires a bent needle compared to the right eye because the angle of the oblique sclerotomy is affected by the nose.

Third, an oblique angled sclerotomy without a horizontal intrascleral tunnel was used to reduce retinal damage. Yamane et al. described a two-step sclerotomy procedure, which consists of a horizontal sclerotomy and an angled sclerotomy to the sulcus, as shown in the video clip of the operation^19,21^. The needle within the two-step sclerotomy must become parallel to the intrascleral tunnel, when left un-grasped, after entry into the vitreous cavity. Thus, it is possible that the needle may cause retinal injury during or after placement of the leading haptic. An oblique (with an angle of 30º with respect to the limbus) angle (less than 5–10º with respect to the surface of the iris) may reduce the risk of retinal injury because when the needle is not grasped, it does not come into contact considerably with the peripheral retina or ciliary body.

Recently, several studies have described modified methods for intrascleral fixation to reduce retinal injury. Ganne et al. proposed the use of a silicone stopper to temporarily fixate the leading haptic after externalisation of the leading haptic to reduce the risk of peripheral retinal injury due to the first needle before manipulation of the trailing haptic^15^. Hwang et al. and Bonell et al. have also described modified methods^14,22^; specifically, they made and buried the flange of the leading haptic into the intrascleral tunnel before the procedure involving the trailing haptic^14,22^. The prevention of injuries to the ciliary body and peripheral retina is very important. However, placing the leading haptic within the needle makes the insertion of the second haptic easier. Thus, we adjusted the angle of the sclerotomy to 30º with respect to the limbus. When the needle is initially inserted through the oblique sclerotomy or after the leading haptic is placed into the needle, the angle of the needle may not be risky.

Fourth, the leading haptic can be directly threaded into the lumen of the needle without an additional corneal incision, the use of intraocular forceps, or the help of a surgical assistant. Threading with intraocular forceps through the cornel incision is not easy for novice surgeons, as it requires a horizontally angled procedure through the limbal paracentesis. Moreover, the tip of the leading haptic is located at the edge of the AC after IOL insertion in Yamane’s technique. Thus, the threading procedure sometimes causes injury to the iris or pigment dispersion due to the intraocular forceps or the tip of the haptic.

Fifth, trailing haptics can be easily threaded with a one-time use of intraocular forceps using the left hand without additional paracentesis or bi-manual intraocular forceps. The second threading step of the trailing haptic into the second needle is the most challenging step of Yamane’s technique^22,23^. Several authors have described a modified technique for this step. Kim first described the trailing-haptic-first modification^23^. Next, Bonnell et al. proposed the threading procedure performed within the posterior segment^22^. However, if the main corneal incision for IOL injection was made in the 12 o’clock to 1 o’clock clockwise direction (left side of the meridian plane), the procedure using left hand-intraocular forceps could be performed simply without additional paracentesis or additional procedures.

Using this modified technique, intrascleral fixation surgery was easily performed in almost all cases. The average UCVA and BCVA values of the patients significantly improved over the follow-up period; there were no significant complications, and none of the patients required reoperation. However, alternative options should be considered for patients with sunken eyes, eyes with a small lid fissure, and eyes with severe corneal oedema due to an intraoperative PC tear, as it is not easy to perform the surgery in these cases.

We did not experience any case of haptic injury using the 27-gauge needle and the disposable forceps. However, threading into the 30-gauge ultra-thin wall needle using strong forceps such as IOL holding forceps sometimes induced haptic deformity (data now shown). Thus, gentle manipulation to prevent haptic injury is necessary for successful surgical outcomes. Moreover, there were no cases of flange exposure or conjunctival erosion (Fig. 3A, B). The outer diameter of a 27-gauge needle is 400 µm; we believe that making a flange larger than 400 µm would increase the risk of flange exposure outside or disinsertion inside. Thus, we suggest that a 300 µm-flange using about 1.5–2.0 mm of haptic is ideal for the 27-gauge technique (Fig. 3C). Additionally, there were no hypotony cases. We tried to prevent hypotony by suturing all the surgical wounds, because we thought that eyes with hypotony would have a risk of haptic protrusion or exposure. Thus, clear cornea incisions and all sclerotomies that tended to leak were sutured.

Anterior segment OCT was performed to measure the tilt of the optic and to evaluate the intrascleral flanged ending of the haptic in all the eyes following surgery. However, the built-in software did not support the measurement of the angle of the two horizontal lines (the iris and IOL planes). Thus, ImageJ software was used. The IOL tilt was evidently non-significant (Fig. 3D) and postoperative lens induced astigmatism was − 0.20 ± 0.41 dioptres.

Recently, aspherical IOLs have been widely used (more than spherical IOLs) to compensate for the positive spherical aberration of the cornea and to reduce high-order aberration^24,25^. However, tilting of the aspherical IOL can induce more asymmetrical aberration compared with spherical IOL^26,27^. Moreover, extension of the haptic for intrascleral fixation causes an increase in the astigmatic aberration of IOL^28^. Thus, the use of spherical IOLs is thought to be more favourable than aspherical IOLs if both types of three-piece IOLs are available; although, in this study, there was no difference in surgical outcomes between the aspherical and spherical IOLs.

This study had several limitations. First, this was neither a prospective nor a comparative study. Therefore, there may be a selection bias. Second, we performed the surgeries using a 30-gauge needle; however, the number of patients was small, and the follow-up period was less than 12 months. Thus, we could not comparatively analyse these patients. Third, the total number of patients was relatively small, and the follow-up period was short. Nevertheless, none of the cases showed any significant complications. Fourth, intra-scleral fixation techniques have evolved through the pars plana approach because the technique requires an intravitreal approach with vitreoretinal forceps. However, we think that potential readers of this study can modify this technique using the limbal approach to make simpler. Finally, a future prospective study with a well-designed comparison with other scleral fixation surgeries would be more informative.

In conclusion, we proposed several modifications in the instrascleral IOL fixation technique to make the procedure easier and safer, including the use of a 27-gauge needle, non-bent needle, oblique sclerotomy, direct threading of the leading haptic, and one step-easy placement of the following haptic. We believe that these technical modifications will contribute to the widespread use of the intrascleral fixation technique.

## Supplementary Information

Supplementary Information 1.

Supplementary Video 1.

Supplementary Video 2.

Supplementary Video 3.

Supplementary Video 4.

Supplementary Video 5.

Supplementary Video 6.

## Data Availability

The datasets during and/or analysed during the current study are available from the corresponding author on sreasonable request.

## References

[1] Wagoner MD, Cox TA, Ariyasu RG, Jacobs DS, Karp CL (2003). Intraocular lens implantation in the absence of capsular support: a report by the American Academy of Ophthalmology. Ophthalmology.

[2] Hennig A (1997). Randomised controlled trial of anterior-chamber intraocular lenses. Lancet (London, England).

[3] Auffarth GU, Wesendahl TA, Brown SJ, Apple DJ (1994). Are there acceptable anterior chamber intraocular lenses for clinical use in the 1990s? An analysis of 4104 explanted anterior chamber intraocular lenses. Ophthalmology.

[4] Anbari A, Lake DB (2015). Posteriorly enclavated iris claw intraocular lens for aphakia: long-term corneal endothelial safety study. Eur. J. Ophthalmol..

[5] Yen KG, Reddy AK, Weikert MP, Song Y, Hamill MB (2009). Iris-fixated posterior chamber intraocular lenses in children. Am. J. Ophthalmol..

[6] Kodjikian L (2006). Combined pars plana phacofragmentation, vitrectomy, and Artisan lens implantation for traumatic subluxated cataracts. Retina (Philadelphia, Pa.).

[7] Omulecki W, Nawrocki J, Sempinska-Szewczyk J, Synder A (1997). Transscleral suture fixation and anterior chamber intraocular lenses implanted after removal of posteriorly dislocated crystalline lenses. Eur. J. Ophthalmol..

[8] Lanzetta P, Menchini U, Virgili G, Crovato S, Rapizzi E (1998). Scleral fixated intraocular lenses: an angiographic study. Retina (Philadelphia, Pa.).

[9] Helal M, el Sayyad F, Elsherif Z, El-Maghraby A, Dabees M (1996). Transscleral fixation of posterior chamber intraocular lenses in the absence of capsular support. J. Cataract Refract. Surg..

[10] Buckley EG (2007). Hanging by a thread: The long-term efficacy and safety of transscleral sutured intraocular lenses in children (an American Ophthalmological Society thesis). Trans. Am. Ophthalmol. Soc..

[11] Gabor SG, Pavlidis MM (2007). Sutureless intrascleral posterior chamber intraocular lens fixation. J. Cataract Refract. Surg..

[12] Agarwal A (2008). Fibrin glue-assisted sutureless posterior chamber intraocular lens implantation in eyes with deficient posterior capsules. J. Cataract Refract. Surg..

[13] Rodriguez-Agirretxe I, Acera-Osa A, Ubeda-Erviti M (2009). Needle-guided intrascleral fixation of posterior chamber intraocular lens for aphakia correction. J. Cataract Refract. Surg..

[14] Hwang ES, Warren CC, Koenig SB (2018). Flanged intrascleral intraocular lens fixation with a single needle. J. Cataract Refract. Surg..

[15] Ganne, P., Baskaran, P. & Krishnappa, N. C. Re: Yamane et al.: Flanged intrascleral intraocular lens fixation with double-needle technique (Ophthalmology. 2017;124:1136–1142). *Ophthalmology* **124**, e90–e91. 10.1016/j.ophtha.2017.07.007 (2017). 10.1016/j.ophtha.2017.07.007 29157439

[16] Bonnell AC, Mantopoulos D, Wheatley HM, Prenner JL (2019). Surgical technique for sutureless intrascleral fixation of a 3-piece intraocular lens using a 30-gauge needle. Retina (Philadelphia Pa).

[17] Akimoto M, Taguchi H, Takayama K, Nakagawa S, Hiroi K (2015). Intrascleral fixation technique using catheter needles and 30-gauge ultrathin needles: lock-and-lead technique. J. Cataract Refract. Surg..

[18] Janse van Rensburg E, Ryu CL, Vila N, Chen JC (2019). Sutureless intrascleral fixation of intraocular lens through self-sealing sclerotomy wounds using haptic externalization and reinternalization technique. J. Cataract. Refract. Surg..

[19] Yamane S, Sato S, Maruyama-Inoue M, Kadonosono K (2017). Flanged intrascleral intraocular lens fixation with double-needle technique. Ophthalmology.

[20] Kelkar A (2018). Comparison of two modified sutureless techniques of scleral fixation of intraocular lens. Ophthal. Surg. Lasers Imaging Retina.

[21] Yamane S, Inoue M, Arakawa A, Kadonosono K (2014). Sutureless 27-gauge needle-guided intrascleral intraocular lens implantation with lamellar scleral dissection. Ophthalmology.

[22] Bonnell AC, Mantopoulos D, Wheatley HM, Prenner JL (2019). Surgical technique for sutureless intrascleral fixation of a 3-piece intraocular lens using a 30-gauge needle. Retina (Philadelphia, Pa).

[23] Kim DB (2018). Trailing-haptic-first modification of double-needle intrascleral haptic fixation technique. J. Cataract Refract. Surg..

[24] Ohtani, S. *et al.* One-year prospective intrapatient comparison of aspherical and spherical intraocular lenses in patients with bilateral cataract. *Am. J. Ophthalmol.* **147**, 984–989, 989.e981. 10.1016/j.ajo.2008.12.037 (2009). 10.1016/j.ajo.2008.12.037 19285656

[25] Ohtani S, Miyata K, Samejima T, Honbou M, Oshika T (2009). Intraindividual comparison of aspherical and spherical intraocular lenses of same material and platform. Ophthalmology.

[26] Baumeister M, Buhren J, Kohnen T (2009). Tilt and decentration of spherical and aspheric intraocular lenses: Effect on higher-order aberrations. J. Cataract Refract. Surg..

[27] Wang L, Koch DD (2005). Effect of decentration of wavefront-corrected intraocular lenses on the higher-order aberrations of the eye. Arch. Ophthalmol..

[28] Kunita D (2017). Effects of optical diameter of intraocular lenses with intrascleral fixation on higher-order aberrations. BMC Ophthalmol..

