# Supplementary material for: The simply modified intrascleral fixation using round flange (SMURF) technique for intrascleral intraocular lens fixation
Source: Sci Rep. 2021 Feb 16;11:3904. doi: 10.1038/s41598-021-81924-y (PMC7886904; doi:10.1038/s41598-021-81924-y)
Supplement: Supplementary file 1 — Supplementary Information 1. [file 41598_2021_81924_MOESM1_ESM.docx]

The **S**imply **M**odified Intrascleral Fixation **U**sing **R**ound **F**lange (SMURF) Technique for Intrascleral Intraocular Lens Fixation

Soa Kim, MD^1^, Jee Taek Kim, MD, PhD^2,*^

^1^Department of Ophthalmology, Suwon Hospital, Gyeonggi Provincial Medical Center, Suwon, South Korea

^2^Department of Ophthalmology, College of Medicine, Chung-Ang University Hospital, Seoul, South Korea

**Supplementary Dataset List**

Supplementary Video 1. Marking and oblique sclerotomy

Supplementary Video 2. Direct insertion of the leading haptic

Supplementary Video 3. Second oblique sclerotomy

Supplementary Video 4. Insertion of the following haptic

Supplementary Video 5. Externalization of the haptics

Supplementary Video 6. Creation and placement of the flange
